# Experimental infection of H5N1 HPAI in BALB/c mice

**DOI:** 10.1186/1743-422X-4-77

**Published:** 2007-07-27

**Authors:** Vasily A Evseenko, Eugeny K Bukin, Anna V Zaykovskaya, Kirill A Sharshov, Vladimir A Ternovoi, George M Ignatyev, Alexander M Shestopalov

**Affiliations:** 1 State Research Center of Virology and Biotechnology "Vector" of Rospotrebnadzor, Koltsovo, Russia

## Abstract

**Background:**

In 2005 huge epizooty of H5N1 HPAI occurred in Russia. It had been clear that territory of Russia becoming endemic for H5N1 HPAI. In 2006 several outbreaks have occurred. To develop new vaccines and antiviral therapies, animal models had to be investigated. We choose highly pathogenic strain for these studies.

**Results:**

A/duck/Tuva/01/06 belongs to Quinghai-like group viruses. Molecular markers – cleavage site, K627 in PB2 characterize this virus as highly pathogenic. This data was confirmed by direct pathogenic tests: IVPI = 3.0, MLD_50 _= 1,4Log10EID_50_. Also molecular analysis showed sensivity of the virus to adamantanes and neuraminidase inhibitors. Serological analysis showed wide cross-reactivity of this virus with sera produced to H5N1 HPAI viruses isolated earlier in South-East Asia. Mean time to death of infected animals was 8,19+/-0,18 days. First time acute delayed hemorrhagic syndrome was observed in mice lethal model. Hypercytokinemia was determined by elevated sera levels of IFN-gamma, IL-6, IL-10.

**Conclusion:**

Assuming all obtained data we can conclude that basic model parameters were characterized and virus A/duck/Tuva/01/06 can be used to evaluate anti-influenza vaccines and therapeutics.

## Backgound

Influenza A (H5N1) virus now becomes a real threat for humans. Since 1997, when first human case of H5N1 HPAI had been reported, more than 317 people were infected and 191 died [1]. Before 2005 attention was attracted to Thailand, Vietnamese and Indonesian viruses. In the beginning of 2005 outbreak on Quinghai lake occurred [2]. Later "Quinghai-like" viruses spreaded to most part of Russia, European countries and Africa and caused numerous outbreaks. Only in Russia more than 1 million of different species and sorts of poultry died and been slaughtered [3]. Confirmed cases in Azerbaijan, Egypt, Iraq, and Turkey was caused by Quinghai-like viruses. Earlier HPAI viruses were investigated in mice [4,5] and murine models were successively used for reverse genetics made influenza vaccines [6]. It was shown that H5N1 HPAI viruses could have different pathogenicity for mice [7]. Several molecular markers were choused to explain differences. Multibasic cleavage site with 627K in PB2 designate to highly pathogenic phenotype for mice. Also important role of pulmonary cytokines elevation was highlighted [8]. Combination of adaptation for wild waterfowl and high virulence for mammals makes Quinghai-like viruses presumably pandemic. Also, in future, because of ability for rapid spreading for long distances, this group of viruses can appear in North and South America and cause outbreaks.

Human disease caused by HPAI viruses can be characterized as acute viral pneumonia aggravated by ARDS, toxic shock and multiple organ failure. System dysfunction mediated by hypercytokinemia and high viral load [9]. To be ready for new influenza pandemy it is necessary to use animal models, in vaccine and antivirals studies, which most closely reflect human disease. Isolates from FRSI SRC VB "VECTOR" repository which were characterized previously were examined for MLD_50_, molecular markers of pathogenicity, sensitivity to amantadines and neuraminidase inhibitors, to be candidates for murine model. Among the investigated isolates A/duck/Tuva/01/06 has best features to be used.

## Results

### Molecular characteristics

**Figure 1:**
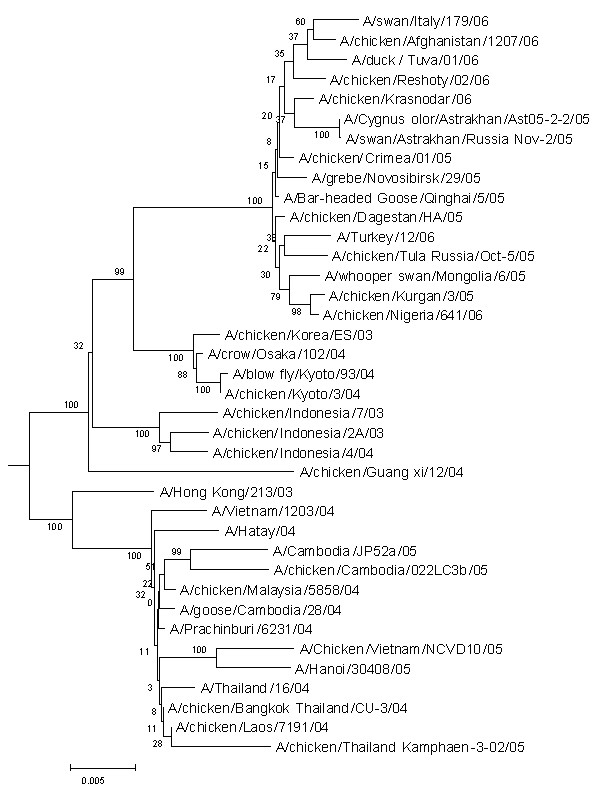
**Some cytokines levels in BALB/c mice sera**. Levels expressed in pg/ml. Mean ± S.D results from 5 mice.

Genes of A/duck/Tuva/01/06 were sequenced and analyzed for molecular markers of pathogenicity. Also phylogenetic analysis was performed. Results are presented in figure 1. A/duck/Tuva/01/06 belongs to group of Qinghai-like viruses. HA contains 5 polybasic aminoacids (PQGRRKKKR↓GL) in cleavege site of HA [15]. The receptor binding domen can be characterized as "avian" [16]. High pathogenicity to mammals in general correlates with presence of 627K in PB2 [17].

The analysis of non-structural protein 1 (NS1) which also could be contributed for high virulence of H5N1 viruses revealed deletion of 5 amino acids similar to those in H5N1 viruses of genotype Z which could be contributed to increased expression of TNF-α and IP-10 protein in primary human macrophages [18]. A/duck/Tuva/01/06 contained Glu_92 _in the NS1 and contained "avian-like" PDZ-domain ligand ESEV [19]. It was shown that the most recent H5N1 strains isolated in Southeast Asia were resistant to amantadine and rimantadine; antiviral drugs targeted the M2 ion channels of influenza A viruses [20,21]. It was also reported about Oseltamivir resistant H5N1 viruses isolation from humans [22,23]. To determine the potential sensitivity of studied H5N1 viruses to these antivirals, amino acid sequences of the M2 and NA proteins were analyzed.

Variants of influenza A viruses resistant to amantadine possessed amino acid substitutions at one of 5 residues (26, 27, 30, 31, and 34) in the M2 protein [24,25]. Sequence analysis did not reveal any mutations associated with resistance to amantadine. Thus all A/duck/Tuva/01/06 is potentially sensitive to this class of antiviral agents. Amino acid residues 119, 274, 292 and 294 in the NA protein (numbering according to the HA of H2 subtype) are crucial for the sensitivity of influenza A viruses to neuraminidase inhibitors [26]; substitution H_274_→Y in the NA conferred resistance to Oseltamivir was observed in clinical H5N1 isolates [25,26]. Sequence comparison of the NA protein of A/duck/Tuva/01/06 aligned with the NA of N2 subtype of A/Wuhan/359/95 (H3N2) influenza virus showed phenotype potentially sensitive to neuraminidase inhibitors.

### Serological features

A/duck/Tuva/01/06 showed wide cross-reactivity with sera against H5N1 HPAI viruses isolated earlier in South-Eastern Asia. HI results can be found in table 1. These features persuade to use this virus in studies of vaccines made from various H5N1 influenza viruses.

**Table 1:** Cross-reactivity of A/duck/Tuva/01/06. Also some other viruses isolated in Russia in 2005–2006 with studied with sera obtained to viruses isolated in South-East Asia previously.

	Polyclonal sera to:					
	Ck/Hidalgo/95	Gs/HK/99	HK/156/97	HK/213/03	VN/1203/04	Prachinbrr/6231/04

Tk/Suzdalka/1–12/05	80	160	10	80	80	20
Ck/Suzdalka/2–6/05	160	320	<10	80	40	10
Gs/Suzdalka/6–10/05	160	320	10	80	80	20
Ck/Omsk/108-14/05	80	160	10	160	80	20
Gray dk/Omsk/105-16/05	160	160	10	160	80	20
mallrd/Dovolnoye/5–26/05	10	10	10	640	320	10
**duck/Tuva/01/06**	**320**	**640**	**40**	**160**	**160**	**40**
Ck/krasnodar/06/06	320	1280	40	320	320	40
Ck/Reshoty/2/06	160	640	40	320	320	80
Gs/Krasnoozerskoye/627/05	160	160	<10	80	80	10

### Animal studies

**Figure 2:**
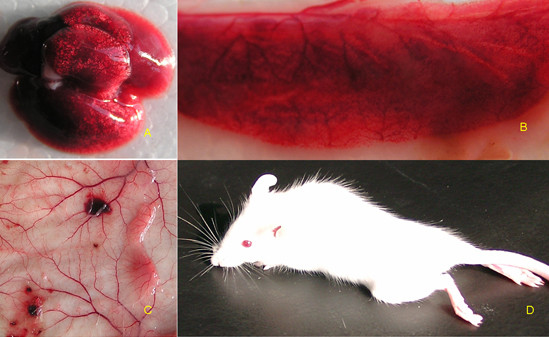
**Phylogenetic tree based on full length sequencesof HA**. Nucleotide sequences were analyzed by using the neighbor-joining method with 500 bootstraps. The phylogenetic tree was rooted to the HA gene of A/goose/Guangdong/1/96 (H5N1) virus.

First MID_50 _and MLD_50 _for A/duck/Tuva/01/06 were determined (table 2). To determine mean time to death (m.t.d) and S.D. we perform four independent experiments. Dose 5MLD_50 _was chosen to get 90–100% mortality rates. Disease can be characterized as violent. Within third and fourth days p.i. all mice demonstrated severe sickness with ruffling of the fur, anorexia and rapid weight loss (data not showed). Also we observed lack of motion activity, group forming. To day 6 mice showed breathlessness, cyanosis and in common – transition to terminal condition. In case of infection by 5MLD_50 _m.t.d. was 8,19 ± 0,18 days. Animals which live till 8–9 days usually had paralyses and paresises (figure 2D, ARDS and figure 2A). Also several atypical manifestations in infected mice were occured during the duration of the experiment. We observed several cases of acute delayed hemorrhagic syndrome with visible intestinal (3 animals totally), intracutaneous hemorrhages (4 animals totally), see figures 2B and 2C. In several cases (9 animals totally) the disease was complicated by severe intestine atony, which can independently lead to death or by pressuring on diaphragm can intensify respiratory failure.

**Table 2:** Pathogenicity and replication of A/duck/Tuva/01/06 in BALB/c mice. EID_50_, 50% egg infectious dose; MID_50_, 50% mouse infectious dose; MLD_50_, 50% mouse lethal dose.

**Virus**	**Log_10 _EID_50_/ml**	**MID_50_†**	**MLD_50_†**	**Organ tissues‡**				
				
				**Lungs**	**Spleen**	**Brain**	**Liver**	**Kidney**
A/duck/Tuva/01/06				5,3 ± 0,5	<1	3,4 ± 0,3	<1	<1

†MID_50 _and MLD_50 _are expressed as number of EID_50_. ‡Mean ± SD from 3 mice, expressed as log_10 _EID50/100 mg of organ tissue.

We also determined virus titers in several organ tissues. As it was expected the highest titers was observed in lungs – 5,3 log EID_50_. Brain titers were also high – 3,4 log EID_50_. In spleen, liver and kidney tissues virus titers were lower then 1 logEID_50 _and considered not significant.

### Cytokines

**Figure 3:**
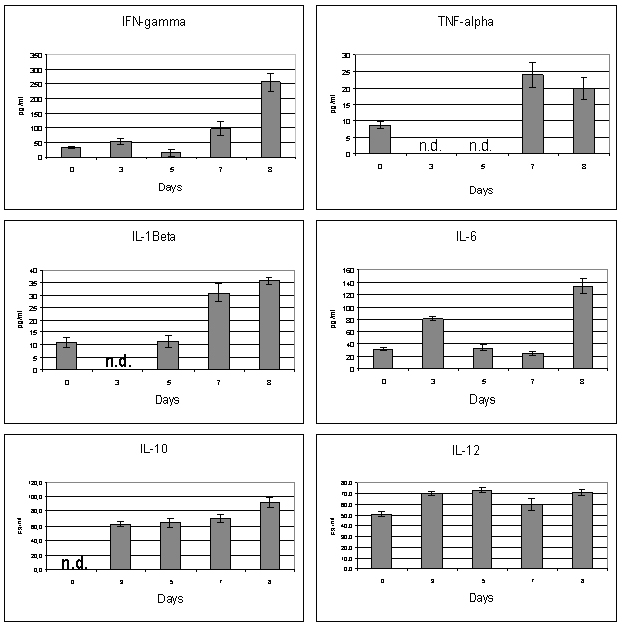
**Gross pathology of BALB/c mice infected with A/duck/Tuva/01/06**. (A) Lungs, (B) Small intestine hemorrhages, (C) intracutaneous hemorrhages, (D) Lower extremities paresis.

We investigated the involvement of several cytokines in immunopathogenesis of experimental H5N1 HPAI infection in mice. Results of ELISA technique revealed alteration of expression both pro-inflammatory and anti-inflammatory cytokines after the challenge (figure 3). In general, the most marked changes of cytokine levels were observed before the death of mice.

The minimal concentration of IFN-γ was detected on day 5 (14.3 ± 10.8 pg/ml), however, its levels enlarged about 8-fold (256 ± 27 pg/ml) during the course of the infection when compared with uninfected animals. On days 3 and 5 systemic production of TNF-α was below the detection limit of the assay. A peak was reached on day 7 by the cytokine (24 ± 3.2 pg/ml) and its levels remained elevated on day 8. Interestingly, concentrations of IL-1β in mice after the challenge were significantly lower in comparison with the constitutive expression of the mediator in intact animals. An abrupt decrease of IL-1β was detected on day 3 post infection, but was followed by step increase from day 5. After the 2.5-fold enlargement on day 3 the levels of IL-6 decreased dramatically on day 5, and the highest levels of the cytokine were determined at the end of observation period (133 ± 12 pg/ml). The constitutive production of IL-10 was undetectable. The dynamics of IL-10 showed a gradual growth with the maximum level (92.1 ± 6.0 pg/ml) reached before the death of mice. We observed statistically significant increase of IL-12 after the challenge. Concentrations of the cytokine retained constant in infected mice, except the unexpected decline occurred on day 7. The expression of IL-18 could not be detected throughout the entire period of observation.

## Discussion

Until 2005 avian influenza was regional problem of several Asian countries. It becomes endemic in Vietnam, Indonesia, Laos, Cambodia and South part of China. Main way of spreading was life poultry markets and later after quarantine measures establishment life birds smuggling becomes one of the main ways. Even if H5N1 HPAI could appear with chicken meet or life birds, dissemination of virus would be stopped by strait quarantine measures. But in 2005 completely adapted to wild waterfowl virus appeared in Quinghai province of China and rapidly speeded. In Russia 9 outbreaks among wild birds were reported [4] and question "why had only some wild waterfowl died?" is still unclear. Most of the outbreaks in Russia associated with wild birds. The same time viruses adapted to wild birds are extremely pathogenic for poultry and mice. This "competitive advantage" makes Quinghai-like viruses most probable candidate to be precursor for new pandemic influenza virus. At the same time pathogenesis of different (phylogenetical clades) HPAI reveal common causes. The principal causes of rapid mice death after infecting with HPAI are primary viral pneumonia, ARDS, lesions of central nervous system and multiple organ failure. Our data suggest that A/duck/Tuva/01/06 strain of HPAI caused lethal pneumonia and spread systemically to the brain in BALB/c mice. Lesion of respiratory epithelium and following an activation of monocytes/macrophages results in a release of proinflammatory cytokines (TNF-α, IL-6) which are a hallmark of ARDS in murine model [27]. Despite powerful anti-influenza virus effects of TNF-α in lung tissue, as it was described previously [28], we consider that elevated production of the cytokines seems to be crucial in the pathogenesis of HPAI infection. Moreover, it was shown that lethal H5N1 viruses are resistant to antiviral effects of interferons and TNF-α [29]. Virus-induced overexpression of TNF-α as well as high IFN-γ lead to activation of endothelium and imbalance in blood coagulation system [30]. This may explain the hemorrhagic syndrome as observed in some of animals. To pay attention that IL-12 is a potent inducer of IFN-γ synthesis by blood mononuclear cells [31], we concluded the same cytokines hyperproduction reflects macrophage overactivation and subsequent hypercytokinemia. This cascade of events including inflammatory mediator production, changes in blood coagulation system and microvascular permeability was denoted as systemic inflammatory response syndrome (SIRS) [32]. On the other hand, we proposed that the prominent production of IL-10 from the early stages of the experimental HPAI infection was the compensatory response to overproduction of proinflammatory cytokines such as TNF-α, IL-6 and IL-12. However, the role of IL-10, which principle function seems to be containment and eventual termination of inflammation [33], in HPAI pathogenesis is unclear. Also there is an uncertain discrepancy between undetectable expression of IL-18 and high levels of other Th1-cytokines (IFN-γ and IL-12).

Summing up, in our study BALB/c mice infected with HPAI, strain A/duck/Tuva/01/06, appeared to be able to produce the innate immune response, which culminated to the development of shock and subsequent multiple organ failure. The main characteristics of our model are comparable to the previously described fatal cases of H5N1 influenza in humans [10,11]. Proposed model reflects lesions not only same organs but also mediating levels of some (IFN-γ, IL-6, IL-10) cytokines in terminal conditions.

The implication of different cytokines in immunopathogenesis of experimental HPAI is beyond question. But to understand exact mechanisms, which determine the disease outcome, further experiments remain to be done.

## Conclusion

A/duck/Tuva/01/06 belongs to Quinghai-like group viruses. Molecular markers – cleavage site, K627 in PB2 characterize this virus as highly pathogenic. This data was confirmed by direct pathogenic tests: IVPI = 3.0, MLD_50 _= 1,4EID_50_. Also molecular analysis showed sensivity of the virus to adamantanes and neuraminidase inhibitors. Serological analysis showed wide cross-reactivity of this virus with sera produced to H5N1 HPAI viruses isolated earlier in South-East Asia. Mean time to death of infected animals was 8,19 ± 0,18 days. First time acute delayed hemorrhagic syndrome was observed in mice lethal model. Hypercytokinemia was determined by elevated sera levels of IFN-γ, IL-6, IL-10. Assuming all obtained data we can conclude that basic model parameters were characterized and virus A/duck/Tuva/01/06 can be used to evaluate anti-influenza vaccines and therapeutics.

## Materials and methods

All experiments were performed in BSL 3+ facilities of FSRI SRC VB "Vector" of Rospotrebnadzor licensed for working with highly pathogenic avian influenza viruses.

Stock of A/duck/Tuva/01/06 was produced in 9 days-old chicken embryos. Allantoic fluid was aliquoted and stored at -80°C. The infectivity of stock viruses was determined in 10 days-old embryonated chicken eggs; titers were calculated by the method of Reed and Muench [10] and were expressed as log_10 _of 50% egg infective dose (EID_50_) in 1 ml of allantoic fluid.

### Viral RNA isolation RT-PCR and Sequencing

Viral RNA was isolated from virus-containing allantoic fluid with the RNeasy Mini kit (Qiagen, Valencia, CA) as specified by the manufacturer. Uni-12 primer was used for reverse transcription. PCR was performed with a set of primers specific for each gene segment of Influenza A virus [11]. PCR products were purified with the QIAquick PCR purification (Qiagen).

Sequencing was done with Beckman Coulter GenomeLab™ Methods development kit Dye terminator Cycle Sequencing according instructions of manufacturer. Primers for sequence were obtained from E. Hoffman (SJCRH, Memphis, TN). Sequence products were analyzed on automatic sequence analyzer Beckman Coulter CEQ2000.

### Phylogenetic Analysis

Phylogenetical analysis was done on HA full gene sequence DQ861291 using MEGA 2.1 software. Phylogenetical tree was built by Neighbor-Joining method; matrix of distances was counted with p-distance algorithm. Reliability of clades was checked with bootstrap analysis with 500 replications. Other genes in GenBank DQ861291–DQ861295.

### Serological characterization

Cross-reaction of A/duck/Tuva/01/06 was defined by hemagglutination inhibition test (HI) with 0.5% CRBC [12] with a panel of antisera against H5N1 HPAI.

### Animal Studies

Six-week-old inbred male BALB/c mice (vivarium of FRSI SRC VB "Vector"). Animals were placed to individual cages with food and water available *ad libitum*. To determine the MLD_50 _and MID_50_, mice were anaesthetized by diethyl ether inhalation and infected intranasally with 50 μl 10-fold serial dilutions of allantoic fluidin PBS (pH 7,2). Each group contained 10 animals. Animals were observed daily for 15 days for mortality (MLD_50_) or sacrificed on day 5 after the challenge with following virus detection in the lungs by inoculation of 10 days-old embryonated chicken eggs (MID_50_). MLD_50 _and MID_50 _were calculated by the method of Reed and Muench. Animals from group where 1MLD_50 _had been observed were taken to determine virus titers in lung, spleen, kidneys, and liver and brain tissues. Mind time to death (m.t.d) was calculated as previously described [13]. Pathogenicity to chickens was determined by IVPI test [14]. All animal studies were performed according protocols approved by Animal Care & Use committee of FSRI SRC VB "Vector".

### Cytokines

To determine IFN-γ, TNF-α, IL-6, IL-10, IL1-β, IL-12 we use ELISA R& D Systems kits (Minneapolis, MN, USA). Serum levels of IL-18 were measured using commercial Mouse IL-18 ELISA test kit (MBL, Nagoya, Japan). Detection limits were as follows: TNF-α, less then 5,1 pg/ml; IL1-β, 3,0 pg/ml; IL6, 3,1 pg/ml; IL10, 4,0 pg/ml; IL-18, 25 pg/ml. Sera was taken on 0,3,5,7,8 days and aliquots and stored -80°C upon usage. Day 8 was chosen because m.t.d defined earlier in the work was 8,19 ± 0,18 days. Statistics was performed with Student t-test. Values p < 0,05 considered to be reliable.

## Competing interests

The author(s) declare that they have no competing interests.

## Authors' contributions

VE carried out molecular genetic analysis, performed animal studies, design of experiments and drafted manuscript. EB performed immunoassays and obtained data analysis. AZ participated in animal studies. KS assisted in animal studies. VT was responsible for sequence. GI participated in study design and coordination. AS carried out coordination. All authors read and approved the final manuscript.

## References

[B1] World Health Organization Epidemic and Pandemic Alert and Response (EPR). Avian influenza.

[B2] Liu J, Xiao H, Lei F, Zhu Q, Qin K, Zhang Xw, Zhang Xl, Zhao D, Wang G, Feng Y, Ma J, Liu W, Wang J, Gao GF HighlyPathogenic H5N1 Influenza Virus Infection in Migratory Birds. Science.

[B3] Onishchenko GG (2006). Avian influenza in Siberia – 2005: Laboratory and epidemiological studies, antiepidemic measures during the epizooty of avian influenza in poultry in Siberian and Ural Federal Regions of the Russian Federation (July – November 2005).

[B4] Lipatov AS, Krauss S, Guan Y, Peiris M, Rehg JE, Perez DR, Webster RG (2003). Neurovirulence in mice of H5N1 influenza virus genotypes isolated from Hong Kong poultry in 2001. J Virol.

[B5] Maines TR, Lu XH, Erb SM, Edwards L, Guarner J, Greer PW, Nguyen DC, Szretter KJ, Chen LM, Thawatsupha P, Chittaganpitch M, Waicharoen S, Nguyen DT, Nguyen T, Nguyen HH, Kim JH, Hoang LT, Kang C, Phuong LS, Lim W, Zaki S, Donis RO, Cox NJ, Katz JM, Tumpey TM (2005). Avian influenza (H5N1) viruses isolated from humans in Asia in 2004 exhibit increased virulence in mammals. J Virol.

[B6] Lipatov AS, Webby RJ, Govorkova EA, Krauss S, Webster RG Efficacy of H5 influenza vaccines produced by reverse genetics in a lethal mouse model. J Infect Dis.

[B7] Maines TR, Lu XH, Erb SM, Edwards L, Guarner J, Greer PW, Nguyen DC, Szretter KJ, Chen LM, Thawatsupha P, Chittaganpitch M, Waicharoen S, Nguyen DT, Nguyen T, Nguyen HH, Kim JH, Hoang LT, Kang C, Phuong LS, Lim W, Zaki S, Donis RO, Cox NJ, Katz JM, Tumpey TM (2005). Avian influenza (H5N1) viruses isolated from humans in Asia in 2004 exhibit increased virulence in mammals. J Virol.

[B8] Lipatov AS, Andreansky S, Webby RJ, Hulse DJ, Rehg JE, Krauss S, Perez DR, Doherty PC, Webster RG, Sangster MY (2005). Pathogenesis of Hong Kong H5N1 influenza virus NS gene reassortants in mice: the role of cytokines and B- and T-cell responses. J Gen Virol.

[B9] de Jong MD, Simmons CP, Thanh TT, Hien VM, Smith GJ, Chau TN, Hoang DM, Van Vinh Chau N, Khanh TH, Dong VC, Qui PT, Van Cam B, Ha DQ, Guan Y, Peiris JS, Chinh NT, Hien TT, Farrar J Fatal outcome of human influenza A (H5N1) is associated with high viral load and hypercytokinemia. Nat Med.

[B10] Reed LJ, Muench H (1938). A simple method for estimating fifty percent endpoints. Am J Hyg.

[B11] Hoffmann E, Stech J, Guan Y, Webster RG, Perez DR (2001). Universal primer set for the full-length amplification of all influenza A viruses. Arch Virol.

[B12] Palmer DF, Dowdle MT, Coleman MT, Schild GC (1975). In: Advanced laboratory techniques for influenza diagnosis. (1975) US Department of Health, Education and Welfare Immunology Series 6.

[B13] Ashmarin IP, Vorobyov AA (1962). Statistical methods in microbiological studies.

[B14] Capua I, Mutinelli F (2001). A color atlas and text on avian influenza. Papi Editore, Bologna, Italy.

[B15] Lipatov AS, Govorkova EA, Webby RJ, Ozaki H, Peiris M, Guan Y, Poon L, Webster RG (2004). Influenza: emergence and control. J Virol.

[B16] Stevens J, Blixt O, Tumpey TM (2006). Structure and receptor specificity of the hemagglutinin from an H5N1 influenza virus. Science.

[B17] Salomon R, Franks J, Govorkova EA, Ilyushina NA, Yen HL, Hulse-Post DJ, Humberd J, Trichet M, Rehg JE, Webby RJ, Webster RG, Hoffmann E (2006). The polymerase complex genes contribute to the high virulence of the human H5N1 influenza virus isolate A/Vietnam/1203/04. J Exp Med.

[B18] Guan Y, Poon LL, Cheung CY, Ellis TM, Lim W, Lipatov AS, Chan KH, Sturm-Ramirez KM, Cheung CL, Leung YH, Yuen KY, Webster RG, Peiris JS (2004). H5N1 influenza: a protean pandemic threat. ProcNatl Acad Sci USA.

[B19] Obenauer JC, Denson J, Mehta PK, Su X, Mukatira S, Finkelstein DB, Xu X, Wang J, Ma J, Fan Y, Rakestraw KM, Webster RG, Hoffmann E, Krauss S, Zheng J, Zhang Z, Naeve CW (2006). Large-scale sequence analysis of avian influenza isolates. Science.

[B20] Li KS, Guan Y, Wang J, Smith GJ, Xu KM, Duan L, Rahardjo AP, Puthavathana P, Buranathai C, Nguyen TD, Estoepangestie AT, Chaisingh A, Auewarakul P, Long HT, Hanh NT, Webby RJ, Poon LL, Chen H, Shortridge KF, Yuen KY, Webster RG, Peiris JS (2004). Genesis of a highly pathogenic and potentially pandemic H5N1 influenza virus in eastern Asia. Nature.

[B21] Ilyushina NA, Govorkova EA, Webster RG (2005). Detection of amantadine-resistant variants among avian influenza viruses isolated in North America and Asia. Virology.

[B22] Le QM, Kiso M, Someya K, Sakai YT, Nguyen TH, Nguyen KH, Pham ND, Ngyen HH, Yamada S, Muramoto Y, Horimoto T, Takada A, Goto H, Suzuki T, Suzuki Y, Kawaoka Y (2005). Avian flu: isolation of drug-resistant H5N1 virus. Nature.

[B23] de Jong MD, Tran TT, Truong HK, Vo MH, Smith GJ, Nguyen VC, Bach VC, Phan TQ, Do QH, Guan Y, Peiris JS, Tran TH, Farrar J (2005). Oseltamivir resistance during treatment of influenza A (H5N1) infection. N Engl J Med.

[B24] Hay AJ, Wolstenholme AJ, Skehel JJ, Smith MH (1985). Themolecular basis of the specific anti-influenza action of amantadine. EMBO J.

[B25] Pinto LH, Holsinger LJ, Lamb RA (1992). Influenza virus M2protein has ion channel activity. Cell.

[B26] Gubareva LV (2004). Molecular mechanisms of influenza virus resistance to neuraminidase inhibitors. Virus Res.

[B27] Xu T, Qiao J, Zhao L, Wang G, He G, Li K, Tian Y, Gao M, Wang J, Wang H, Dong C (2006). Acute Respiratory Distress Syndrome Induced by Avian Influenza A (H5N1) Virus in Mice. Am J Respir Crit Care Med.

[B28] Seo SH, Webster RG (2002). Tumor necrosis factor alpha exerts powerful anti-influenza virus effects in lung epithelial cells. J Virol.

[B29] Seo SH, Hoffmann E, Webster RG (2002). Lethal H5N1 influenza viruses escape host anti-viral cytokine responses. Nat Med.

[B30] Esmon CT (2005). The interactions between inflammation and coagulation. Br J Haematol.

[B31] Gately MK, Wolitzky AG, Quinn PM, Chizzonite R (1992). Regulation of human cytolytic lymphocyte responses by interleukin-12. Cell Immunol.

[B32] Bone RC (1992). Toward an epidemiology and natural history of SIRS (systemic inflammatory response syndrome). Jama.

[B33] Moore KW, Malefyt de Waal R, Coffman RL, O'Garra A (2001). Interleukin-10 and the interleukin-10 receptor. Annu Rev Immunol.

